# Building the evidence base for the economic value of digital health interventions: examples of methods and metrics and opportunities for future directions

**DOI:** 10.1093/oodh/oqae042

**Published:** 2024-12-02

**Authors:** Tara Herrick, Peder Digre, Sarah Skye Yoden

**Affiliations:** Market Dynamics, PATH, 2201 Westlake Avenue, Suite 200, Seattle, WA, 98121, USA; Market Dynamics, PATH, 2201 Westlake Avenue, Suite 200, Seattle, WA, 98121, USA; Department of Health Policy and Management, Harvard University, 677 Huntington Avenue, Boston, MA, 02115, USA

**Keywords:** digital health, digital health interventions, health impact, health outcomes, cost, cost-effectiveness

## Abstract

Quantifying the costs, impact and cost-effectiveness of digital health interventions (DHIs) presents both technical and logistical challenges, largely due to unclear causal pathways to health impact and the tendency of DHIs to be implemented as just one component of an intervention. Few publications provide peer-reviewed estimates of these metrics for DHIs, especially in low- and middle-income countries (LMICs). Yet decision-makers need this evidence to make informed decisions and investments about DHIs. The special collection outlined here offers seven practical and diverse examples of how digital health implementers have assessed the costs and impacts of DHIs in LMICs. These examples can serve as building blocks to inform and guide the development of critically needed methodological approaches for measuring the cost, impact and value of DHIs in a standardized way.

**RESUMEN:**

Cuantificar los costos, el efecto y la efectividad de costos de las intervenciones digitales de salud (DHI, por sus siglas en inglés) representa desafíos tanto técnicos como logísticos, en gran medida debido a la falta de claridad respecto a las secuencias causales para el efecto sobre la salud y la tendencia de las DHI para implementarse como solo un componente de una intervención. Pocas publicaciones ofrecen estimados con revisión por pares de estas métricas para las DHI, especialmente en países de ingresos bajos y medios (LMIC, por sus siglas en inglés). Pero las personas encargadas de tomar decisiones necesitan esta evidencia para tomar decisiones informadas y hacer inversiones relacionadas con las DHI. La colección especial aquí descrita ofrece siete ejemplos prácticos y diversos sobre cómo los ejecutores de salud digital han evaluado los costos y los efectos de las DHI en LMIC. Estos ejemplos pueden usarse como bloques de construcción para informar y guiar el desarrollo de enfoques metodológicos altamente necesarios para medir el costo, el efecto y el valor de las DHI de manera estandarizada.

**RESUMO:**

Quantificar os custos, o impacto e a relação custo-eficácia das intervenções de saúde digitais (ISD) apresenta desafios técnicos e logísticos, em grande parte devido a vias causais pouco claras para o impacto na saúde e à tendência das ISD para serem implementadas como apenas um componente de uma intervenção. Poucas publicações fornecem estimativas revistas por pares destas métricas para as IDS, especialmente nos países de baixo e médio rendimento (PBMR). No entanto, os decisores precisam destas provas para tomar decisões e investimentos informados sobre as ISD. A coleção especial aqui delineada oferece sete exemplos práticos e diversificados de como os implementadores de saúde digital avaliaram os custos e os impactos das ISD nos PBMR. Estes exemplos podem servir como blocos de construção para informar e orientar o desenvolvimento de abordagens metodológicas extremamente necessárias para medir o custo, o impacto e o valor das IDS de forma padronizada.

**RÉSUMÉ:**

La quantification des coûts, de l’impact et de la rentabilité des interventions de santé numérique (ISN) présente des défis à la fois techniques et logistiques, en grande partie en raison du manque de clarté sur les causes contribuant aux impacts sur la santé et de la tendance des ISN à être mises en œuvre comme un simple composant d’une intervention. Peu de publications fournissent des estimations évaluées par des pairs de ces mesures pour les ISN, en particulier dans les pays à revenu faible et intermédiaire (PRFI). Pourtant, les décideurs ont besoin de ces preuves pour prendre des décisions éclairées et réaliser des investissements en matière d’ISN. La collection spéciale présentée ici offre sept exemples pratiques et divers de la manière dont les responsables de la mise en œuvre de la santé numérique ont évalué les coûts et les impacts des ISN dans les PRFI. Ces exemples peuvent servir de base pour informer et guider le développement d’approches méthodologiques indispensables pour mesurer le coût, l’impact et la valeur des ISN de manière standardisée.

A growing number of public health leaders are digitalizing their health systems. Central to digitalization efforts are digital health interventions (DHIs), defined by the World Health Organization (WHO) as ‘a discrete technology functionality—or capability—designed to achieve a specific objective addressing a health system challenge’ [1]. Unfortunately, quantifying the costs, impact or value (i.e. impact achieved for a given cost, such as cost-effectiveness) is a challenge, and few publications provide peer-reviewed estimates of these metrics for DHIs—especially in low- and middle-income countries (LMICs). Several challenges exist in estimating health impact metrics:

1. The impact that the DHIs generate may not be quantifiable using metrics that are standard for drugs, diagnostics and medical devices—such as cost per outcome averted [e.g. case, death or disability-adjusted life year (DALY)] due to the lack of a direct causal pathway to health impact.
2. Some DHIs (e.g. clinical decision support tools) may provide benefits across multiple health areas. This requires quantifying and then aggregating the health impacts for each health area, which is complex and costly.
3. DHIs may serve a critical function to the underlying digital health system infrastructure (e.g. health information exchanges) but quantifying and conceptualizing their contribution to health system improvements and health outcomes is challenging.
4. DHIs are often implemented as part of a broader intervention package, meaning the impact attributable to the DHI alone is difficult to ascertain.
5. DHIs may target indirect contributors to health outcomes, such as efficiency gains for service delivery, rather than directly supporting clinical interactions, making it challenging to assess their direct impact.

**Table 1:** Overview of articles included in the special collection

Authors	Health area	Country	DISAH classification	Focus			Key metric(s)
				Cost	Impact	Value	
Feldacker et al. [2]	N/A	Malawi	2.2. person-centered health records		X		• time
Finnegan et al. [3]	malaria	Kenya	3.1. human resource management		X		• cases averted • deaths averted
Harnisher et al. [4]	N/A	Kenya	2.8. healthcare provider training		X		• number of referrals
Hürlimann et al. [5]	child health	Somalia	2.3. healthcare provider decision support		X		• proportion receiving appropriate treatment • proportion receiving appropriate diagnosis • proportion receiving appropriate prevention measures
Kiruthu-Kamamia et al. [6]	HIV	Malawi	2.5. healthcare provider communication			X	• incremental cost per patient retained
Ruiz-Gaona et al. [7]	N/A	Nigeria	2.5. healthcare provider communication 2.8. healthcare provider training 3.2. supply chain management	X	X		• business performance rate of counterfeit medicines • client satisfaction • size of client base • knowledge of COVID-19 • cost
Winters et al. [8]	malaria	Zambia	4.3. geo spatial information management			X	• cost per case averted

Cost analysis for digital health also presents challenges:

1. Many DHIs classified as global goods are open-source, and users of open-source software often perceive the cost of using the software as free. However, adapting DHIs to local contexts and integrating them into local systems can require substantial time and effort from personnel, as well as additional costs for equipment and licensing. These costs can be unanticipated and difficult to estimate.
2. In circumstances where cost analyses are conducted, ongoing operating costs to maintain the DHIs are frequently excluded, threatening the long-term sustainability of solutions.
3. DHIs rely on substantial human, physical and digital infrastructure, and there is currently no globally adopted standard method for quantifying these dependencies, incorporating them into costing efforts, and analyzing how costs vary when infrastructure availability varies across contexts.

Despite these challenges, quantifying the value of DHIs is important to build alignment across stakeholders on which DHIs will likely provide the highest value to communities and when to scale them across national or subnational contexts. There is demand for more quantitative evidence on the impact of DHIs from both digital health donors and country decision-makers, as illustrated in *Beyond the hype: a call for comprehensive evidence on the economic and health outcomes of digital and data technologies* [9].

The special collection, *Health impact, efficiency gains, costs, and savings from digital health interventions*, helps address this lack of evidence by offering seven practical examples of how digital health implementers have assessed the cost and impact of DHIs in LMICs (Table 1). The collection highlights the diverse range of outcomes and the cross-cutting value that DHIs can achieve. The examples included in the published articles span various health areas, countries, WHO classifications of digital interventions, services and applications in health (DISAH), as well as quantified benefits. These examples also illustrate the challenges of quantifying cost, impact and value in a consistent manner across health areas and DISAH classifications. In some cases, direct or estimated measurement of health impact (e.g. cases averted) was possible, but most outcomes were intermediate outcomes (e.g. number of referrals) that are assumed to be associated with better health outcomes. Nonetheless, the quantification of outcomes—including intermediate outcomes—provides valuable information for decision-makers and donors.

While the diversity of outcomes reported in this special collection and the resources currently available to digital health implementers are encouraging, these examples and resources also reveal the lack of a standardized metric for comparing different DHIs to determine the best value for money. There is a critical opportunity to develop new methods and/or metrics to capture the full range of health outcomes attributable to DHIs. Standardized methods and metrics would allow the production of more consistent and comparable estimates of the cost, impact and value of DHIs across health areas and DISAH classifications. Without new methods and metrics, the ability of decision-makers to allocate limited resources to digital (or non-digital) interventions with the best value for money will remain limited.

The special collection helps address the current lack of methodology by providing a new economic evaluation framework from the World Bank that can be used when determining the economic value of DHIs—consisting of five steps: (i) determine the context, (ii) determine the intervention type, (iii) establish the level of complexity, (iv) set the analytic principles and (v) represent the value proposition [10]. The framework aims to facilitate methodological transparency, thereby improving the overall usefulness of economic evaluations of DHIs. In addition to this framework, Supplement Table 1 provides a list of other methodological resources that may be helpful for digital health implementers who wish to quantify the cost, impact and value of DHIs.

Cost, impact and value studies, such as those included in this collection, are critical inputs for supporting sustainability efforts and play a crucial role in helping decision-makers approve large-scale adoption of and funding for DHIs. The studies in this collection can be used as building blocks to inform and guide future methodological approaches to measure the cost, impact and value of DHIs. With these examples in hand, the digital health community can—and should—continue to advocate for data-informed decision-making about DHIs.

## Supplementary Material

Draft_19Sept2024_Supplement_Table_1_oqae042

## Data Availability

No new data were generated or analysed in support of this article.
