# Supplementary material for: Building the evidence base for the economic value of digital health interventions: examples of methods and metrics and opportunities for future directions
Source: Oxf Open Digit Health. 2024 Dec 2;2(Suppl 2):ii1–3. doi: 10.1093/oodh/oqae042 (PMC11936316; doi:10.1093/oodh/oqae042)
Supplement: Draft_19Sept2024_Supplement_Table_1_oqae042 [file Draft_19Sept2024_Supplement_Table_1_oqae042.docx]

**Building the evidence base for the economic value of digital health interventions: examples of methods and metrics and opportunities for future directions**

Tara Herrick^1^, Peder Digre^1^, Sarah Skye Gilbert^2^

^1^PATH, Market Dynamics, PATH, Seattle, WA, 98121, USA

^2^Harvard University, Health Policy, Cambridge, MA, 02138, USA

**Correspondence:**

Tara Herrick
PATH, Market Dynamics
2201 Westlake Ave, Seattle, WA 98121
therrick@path.org

Supplement Table 1. Resources to estimate cost, impact, and value of digital health interventions.

| **Resource** | **Organization(s)** | **Year** | **Type** | **Focus** | | | **Key considerations** |
| --- | --- | --- | --- | --- | --- | --- | --- |
|  |  |  |  | *Cost* | *Impact* | *Value* |  |
| Framework for the economic evaluation of digital health interventions^[[1]](#footnote-1)^ | World Bank *Wilkinson et al.* | 2024 | Guide |  |  | X | - This article provides a structure for making decisions about the scope of value assessments for digital health interventions (DHIs). - Includes rich literature review of evidence base in digital health as well as set of broader resources for economic evaluations of health interventions beyond digital health area. |
| [Total Cost of Ownership Model](https://dimagi.com/resources/total-cost-ownership/) | Dimagi | 2011 | Tool | X |  |  | - This resource is an Excel-based costing tool to estimate the total cost of adopting CommCare or other mobile technologies. |
| [OneHealth Tool](https://www.who.int/tools/onehealth) | United Nations Inter-Agency Working Group on Costing | 2012 | Tool | X | X |  | - The OneHealth Tool estimates the cost and impact of scaling up health system interventions in low- and middle-income countries (LMICs) but does not yet include module for digital costs. - Users can use the OneHealth Tool for DHIs if they convert their data on DHIs into inputs solicited by the tool. |
| [Electronic Immunization Registry: Practical Considerations for Planning, Development, Implementation and Evaluation](https://www.paho.org/en/documents/electronic-immunization-registry-practical-considerations-planning-development) | Pan American Health Organization | 2017 | Guide | X |  |  | - Chapter 3 includes a section on estimating costs associated with an electronic immunization registry (EIR). |
| [Guidance for Investing in Digital Health](https://www.adb.org/publications/guidance-investing-digital-health) | Asian Development Bank | 2018 | Guide | X | X |  | - This document includes a proposed process for developing an investment case for digital health, which includes a framework for evaluating the costed impact of different DHIs, supporting the design of a comparative analysis method. |
| [Building Resilient and Inclusive Digital Ecosystems: A Toolkit for Using Digital Payments in Development Programs](https://www.usaid.gov/sites/default/files/2022-05/USAID_Digital_Payments_Toolkit_2020.pdf) | USAID  NetHope | 2020 | Tool | X |  |  | - [Tool 2 from Step 3](https://solutionscenter.nethope.org/assets/collaterals/Step_3_Tool_2.xlsx) is an Excel-based costing tool focused on digital cash payments that allows users to quantify cost estimates based on per-unit price and volumes. - It provides only one aggregate estimate per item rather than annualized costs and does not differentiate costs across implementation phases (i.e., it does not distinguish between one-time and ongoing costs). |
| [Digital Implementation Investment Guide (DIIG): Integrating Digital Interventions into Health Programs](https://www.who.int/publications/i/item/9789240010567) | World Health Organization | 2020 | Guide | X | X |  | - Chapter 7 recommends costing by implementation phase, shares cost drivers, and presents a case study of how this information was used to estimate deployment costs of an electronic immunization registry in Tanzania. - Chapter 8 describes how to establish a logic model to achieve health impact and provides an illustrative logic model for MomConnect in South Africa. |
| [Cost Outcomes Research Methodology](https://digitalsquare.org/s/Market-Analytics_Landing-page_Cost-outcomes-methodology.pdf) | Vital Wave  Digital Square | 2021 | Guide | X | X |  | - This resource provides a framework to conduct cost and health impact research for DHIs. |
| [Total Cost of Ownership (TCO) Tool](https://digitalsquare.org/tco-tool) | Digital Square | 2022 | Tool | X |  |  | - This is an Excel-based tool to support generating budgets for DHIs in LMICs. - The TCO Tool can be used to identify the costs associated with DHIs that are aimed at a large number of last-mile users (e.g. community health workers) as well as DHIs that are tailored towards national-level health system management. - The TCO can be used both retrospectively and prospectively, and captures costs across three implementation phases: development, deployment, and operations. |
| [Lives Saved Tool (LiST)](https://www.livessavedtool.org/) | Johns Hopkins University | 2003 | Tool |  | X |  | - This tool is a mathematical model to estimate the impact of changes in health intervention coverage in LMICs and would be appropriate for use if a DHI affects coverage of an included intervention. - The tool only estimates lives saved of children under five and lives saved related to maternal mortality. It also fails to capture potential improvements in morbidity and other health outcomes. - LiST depends on reliable coverage estimates to calculate the number of lives saved, which can be difficult to estimate. |
| [Monitoring and Evaluating Digital Health Interventions: a Practical Guide to Conducting Research and Assessment.](https://www.who.int/publications/i/item/9789241511766) | World Health Organization | 2016 | Guide |  | X |  | - This resource provides guidance for monitoring and evaluating DHIs and includes an overview of key considerations and decisions to make when choosing a method to measure impact. It focuses on primary data collection for project assessments. |
| [Recommendations on Digital Interventions for Health System Strengthening](https://www.who.int/publications/i/item/9789241550505) | World Health Organization | 2019 | Guide |  | X |  | - This document includes a set of expert-vetted outcomes, outputs, and methods for evaluating the impact of DHIs*.* |

1. Published in this special collection. [↑](#footnote-ref-1)
